# Interleukin (IL)-6 and IL-10 Are Up Regulated in Late Stage *Trypanosoma brucei rhodesiense* Sleeping Sickness

**DOI:** 10.1371/journal.pntd.0003835

**Published:** 2015-06-19

**Authors:** Charles D. Kato, Vincent P. Alibu, Ann Nanteza, Claire M. Mugasa, Enock Matovu

**Affiliations:** 1 School of Bio-security, Biotechnical & Laboratory Sciences, College of Veterinary Medicine, Animal Resources & Bio-security, Makerere University, Kampala, Uganda; 2 College of Natural Sciences, Makerere University, Kampala, Uganda; Universidade Federal de Minas Gerais, BRAZIL

## Abstract

**Background:**

Sleeping sickness due to *Trypanosoma brucei rhodesiense* has a wide spectrum of clinical presentations coupled with differences in disease progression and severity across East and Southern Africa. The disease progresses from an early (hemo-lymphatic) stage to the late (meningoencephalitic) stage characterized by presence of parasites in the central nervous system. We hypothesized that disease progression and severity of the neurological response is modulated by cytokines.

**Methods:**

A total of 55 sleeping sickness cases and 41 healthy controls were recruited passively at Lwala hospital, in Northern Uganda. A panel of six cytokines (IFN-γ, IL1-β, TNF-α, IL-6, TGF-β and IL-10) were assayed from paired plasma and cerebrospinal fluid (CSF) samples. Cytokine concentrations were analyzed in relation to disease progression, clinical presentation and severity of neurological responses.

**Results:**

Median plasma levels (pg/ml) of IFN-γ (46.3), IL-6 (61.7), TGF-β (8755) and IL-10 (256.6) were significantly higher in cases compared to controls (p< 0.0001). When early stage and late stage CSF cytokines were compared, IL-10 and IL-6 were up regulated in late stage patients and were associated with a reduction in tremors and cranioneuropathy. IL-10 had a higher staging accuracy with a sensitivity of 85.7% (95% CI, 63.7%-97%) and a specificity of 100% (95% CI, 39.8%-100%) while for IL-6, a specificity of 100% (95% CI, 47.8%-100%) gave a sensitivity of 83.3% (95% CI, 62.2%-95.3%).

**Conclusion:**

Our study demonstrates the role of host inflammatory cytokines in modulating the progression and severity of neurological responses in sleeping sickness. We demonstrate here an up-regulation of IL-6 and IL-10 during the late stage with a potential as adjunct stage biomarkers. Given that both cytokines could potentially be elevated by other CNS infections, our findings should be further validated in a large cohort of patients including those with other inflammatory diseases such as cerebral malaria.

## Author Summary

Sleeping sickness in east and central Africa is caused by a protozoan parasite, *Trypanosoma brucei rhodesiense*. About 12.3 million people are at a risk of acquiring the disease that is fatal if untreated. The disease progresses from an early stage with trypanosomes in blood and lymph to a late stage in which trypanosomes enter the central nervous system. Variations in disease presentation, progression and severity of the disease have been reported and cytokines have been proposed as potential players. Before treatment is commenced, disease stage has to be ascertained since treatment for the two stages is different. The currently used staging criteria depends on finding trypanosomes in the cerebrospinal fluids (CSF) and elevation in CSF white blood cells however, this has been found to have low sensitivity. We therefore measured plasma and cerebrospinal fluid cytokine concentrations and determined associations with disease presentation, stage progression and severity of the neurological response. Our study shows that concentrations of specific cytokines are elevated in sleeping sickness patients and have a potential to discriminate between patients in early or late stage of infection.

## Introduction

Human African Trypanosomiasis (HAT) or sleeping sickness is caused by extra-cellular protozoan parasites *T*. *b*. *rhodesiense* (East and Southern Africa) and *T*. *b*. *gambiense* (West and central Africa). Although an estimated 12.3 million people are at a risk of developing *T*. *b*. *rhodesiense* disease, the number of new cases has reduced for the past 4 years to below 200 cases per year (range 110–190) [1]. The disease progresses in two stages, the hemo-lymphatic or early stage is characterized by the proliferation of trypanosomes in blood and lymph. The second or late stage is characterized by invasion of trypanosomes in the central nervous system (CNS) and appears after weeks in the typically acute *T*. *b*. *rhodesiense* disease or months in the chronic *T*. *b*. *gambiense* HAT.

Traditionally, *T*. *b*. *rhodesiense* HAT has been classified as acute [2]. Recently a wide spectrum of clinical presentations coupled with differences in disease progression and severity has been observed [3,4]. This diversity in disease spectrum has been attributed to variation in infecting parasite genotypes and host immunogenetics [3,5,6]. Although few studies have been documented about cytokine dysregulation in *T*. *b*. *rhodesiense* HAT patients, it is proposed that cytokines might be key players in HAT inflammatory processes [7]. However, contradictions about the role of specific cytokines during HAT progression have been noted [8,9]. In experimental animal models, high levels of pro-inflammatory cytokines (IFN-γ and TNF-α) have been associated with moderate to severe neuropathy [10]. HAT patients in Eastern Uganda (Tororo) with high IFN-γ concentrations exhibited faster progression to CNS disease with a high frequency of moderate to severe neurological impairment [5]. However, in other studies IFN-γ has been associated with parasite control and resistance to disease [8,11]. The late stage of the disease has been associated with elevated levels of counter inflammatory cytokines in the CNS of both HAT patients and experimental animal models [7]. The few previous studies indicated that IL-10 and IL-6 were up regulated in the late stage and were associated with reduced severity of neuropathology in experimental [10] and natural infections [3,12,13]. However, in another related study, IL-10 and IL-6 were not associated with neurological severity [14]; it was thus necessary to further investigate this controversy as we have done in this study.

Since clinical signs of HAT are not specific, disease staging to guide treatment is based on examination of cerebrospinal fluid (CSF) [15]. Early stage patients are treated with suramin and late stage patients with melarsoprol that is associated with a reactive encephalopathy in 10% of the patients resulting in an overall mortality of 5% [16]. Currently the WHO criteria is recommended for staging [17], with patients having a WBC of ≤ 5 WBC/μl and no trypanosomes in the CSF classified as early stage, while those with greater than 5 WBC/μl or trypanosomes in the CSF are in the late stage. However, for *T*. *b*. *gambiense* there is contradictory information about the effectiveness of treating patients with 6–20 WBC/μl as early stage [18–20]. A number of adjunct biomarkers for late stage including cytokines have been proposed in both experimental animal models and human patients [21–25].

In this study, we hypothesized that stage progression and severity of neuropathology is modulated by host inflammatory cytokines. We used patient clinical data, paired plasma and CSF samples from patients presenting at Lwala hospital in Northern Uganda to analyze a panel of cytokines (IFN-γ, IL1-β, TNF-α, IL-6, TGF-β and IL-10). We further evaluated the potential of CSF cytokines as stage biomarkers.

## Materials and Methods

### Ethical statement

Ethical review for this study was by the Institutional Review Board (IRB) of the Vector Control Division, Ministry of Health; final approval was provided by the Uganda National Council for Science and Technology (UNCST). In all cases, patients recruited in the study were given written and verbal information about the project objectives in local language so as to give written informed consent. For patients below 16 years, consent was given by the legal guardian, but they were also asked for assent. All samples used in the study were remnants from the routine normal diagnostic procedures required to guide treatment.

### Study design

Patients were recruited passively at Lwala hospital, a sleeping sickness referral center in Northern Uganda (Kaberamaido District) between 2012 and 2014. The hospital serves a large catchment area spanning several districts including Kaberamaido, Dokolo, Alebtong, Kole, Lira and Soroti. Within this region, an estimated 7.9 million people are at a risk of developing *T*. *b*. *rhodesiense* sleeping sickness [17] Routine diagnosis of suspected HAT patients, was done by microscopic examination of wet and thick blood films from finger prick blood [15], or using the Heamatocrit Centrifugation Technique [26]. If the blood smear was positive for trypanosomes, or the patient presented with suspicious HAT signs, a lumbar puncture was performed following WHO disease staging guidelines [17]. White blood cell (WBC) counts were done by the Neubauer Haemocytometer. CSF analysis for trypanosomes was performed by the modified single centrifugation method [27]. Late stage infection was confirmed by the presence of trypanosomes in the CSF and/or a white blood cell count > 5/μl. Treatment for all HAT patients followed the recommendations of the WHO [17]; early stage patients were treated with five intravenous injections of suramin every 7 days while late stage patients received melarsoprol over 10 days.

### Clinical examination of patients

A detailed clinical history was sought from each patient. Self-reported disease duration at the time of admission was taken as the period since the patient observed the first HAT related clinical signs. Physical examination was done by a medical officer and both nonspecific HAT signs and neurological involvement recorded. The degree of neurological involvement was assessed using the Glasgow coma scale (GCS) [28]. Patients with a GCS of 13–15 were classified as mild, while those with a score of 9–12 and ≤8 were classified as moderate and severe impairment respectively. Data recorded on the clinical form included demographic characteristic, self-reported symptoms, perceived onset of symptoms, clinical presentation of the disease, laboratory findings, treatment schedule and disease outcome. Neurological symptoms such as convulsions, tremors, urinary incontinence, psychotic behavior and sleep disorders were recorded.

### Cytokine assays

For cytokine assays, a 5ml blood sample was collected from each patient in EDTA vacutainers and centrifuged for 10min at 3000g. For cerebrospinal fluid analysis, 3–4ml were drawn by lumbar puncture. As controls samples, plasma was collected from HAT free individuals consulting at the hospital. Due to ethical considerations, CSF from controls was not obtained. Both plasma and CSF samples were aliquoted and immediately stored in liquid nitrogen until further analysis. Cytokine concentrations (IFN-γ, IL1-β, TNF-α, IL-6, TGF-β and IL-10) were measured in triplicates from paired plasma and CSF using a solid phase sandwich ELISA (OptEIA, Becton Dickinson, Belgium) as previously described [5,29].

### Statistical analysis

Data analysis was done using IBM SPSS version 22 and GraphPad Prism version 6.0 statistical software. Deviation from normality was tested using D'Agostino-Pearson normality test. Because none of the cytokines presented a normal distribution, data was presented as medians. Comparisons between groups were done using the Mann-Whitney U and Kruskal-Wallis non-parametric tests at a significant level (P< 0.05, 2 tailed). Correlation analysis was done using bivariate non-parametric Spearman correlation test set at a significance level of (P< 0.01 and P< 0.05, 2 tailed tests). To determine the potential of cytokines as late stage markers, receiver operator characteristic (ROC) curves were used to calculate the area under the ROC curve (AUC) with sensitivity and specificity predictions for each marker [30]. Sensitivity and specificity calculations were performed in GraphPad Prism version 6.0 statistical software using equations indicated below. Each value in the data set was used as a cut off value and a thresh hold cutoff value selected as that producing the best combination of sensitivity and specificity.

Sensitivity = True Positive/ (True Positive + False Negative)

Specificity = True Negative/ (True Negative + False Positive)

## Results

### Subjects baseline characteristics

A total of 55 patients and 41 healthy controls were recruited passively at Lwala Hospital between 2012 and 2014. The sex-ratio (male: female) was 1:3 with a median age for HAT cases of 20 years (Table 1). Late stage cases were significantly more common (44 out of 54, (P< 0.0001). Disease stage for one patient could not be ascertained due to limited amounts of CSF. During the study period, 1 patient (1.8%) died. Self-reported duration of illness was significantly longer among late stage patients (0.95 months, range 0.25–7, (P< 0.04). The mean CSF white blood cell count before treatment was 33.4±4.9 cells/μl (range 1–204) with trypanosomes demonstrated in CSF of 38 patients (70%). There was no significant difference in the median blood stream parasitemia between early stage (11.2 x10^4^ trypanosomes/ml) and late stage (11.3 x 10^4^ trypanosomes/ml) patients, however blood stream trypanosome numbers were significantly higher than in CSF (P< 0.0001). Malaria co-infections were detected in 6 (11%) HAT cases and subsequently omitted from cytokine and clinical data analysis. The observed nonspecific signs of HAT and neurological signs are presented in Table 1.

**Table 1:** Patient’s baseline characteristics.

Characteristic	Early stage	Late stage	p value
**Disease stage**	10	44 (81.5%)	<0.0001*
**Sex (male/Female)**	7/3	24/20	0.489
**Age (Median)**	18	20	0.48
**Mortality**	0	1 (1.8%)	0.74
**Trypanosomes in CSF**	0	38 (70%)	<0.000*
**Parasitemia/ml**	11.2x10^4^	11.3x10^4^	0.896
**CSF Parasitosis/ml**	0	7x10^4^	<0.000*
**Disease duration (median)**	0.25	0.95	0.04*
**Glasgow Coma Score**			
Mild (13–150)	6	12 (66.7%)	0.238
Moderate (9–12)	0	3	
Severe (≤8)	0	1	
**Clinical presentation**			
Fever	6	43 (87.8%)	1.0
Headache	10	33 (67.3%)	0.09
Chancre	0	1 (2%)	0.56
Edema	0	10 (20.4%)	0.67
Ascites	0	1 (2%)	0.75
Hepatomegaly	0	2 (4.1%)	0.73
Splenomegaly	0	6 (12.2%)	0.144
Lymphadenopathy	2	8 (16.3%)	0.702
Gait abnormalities	0	9 (18.4%)	0.239
Tremors	0	6 (12.2%)	0.285
Urinary incontinence	0	7 (14.3%)	0.145
Cranioneuropathy	0	7 (14.3)	0.288
Somnolence	4	21 (42.9%)	0.141

*Significantly higher in late stage patients.

### Plasma cytokine levels and disease progression

**Fig 1:**
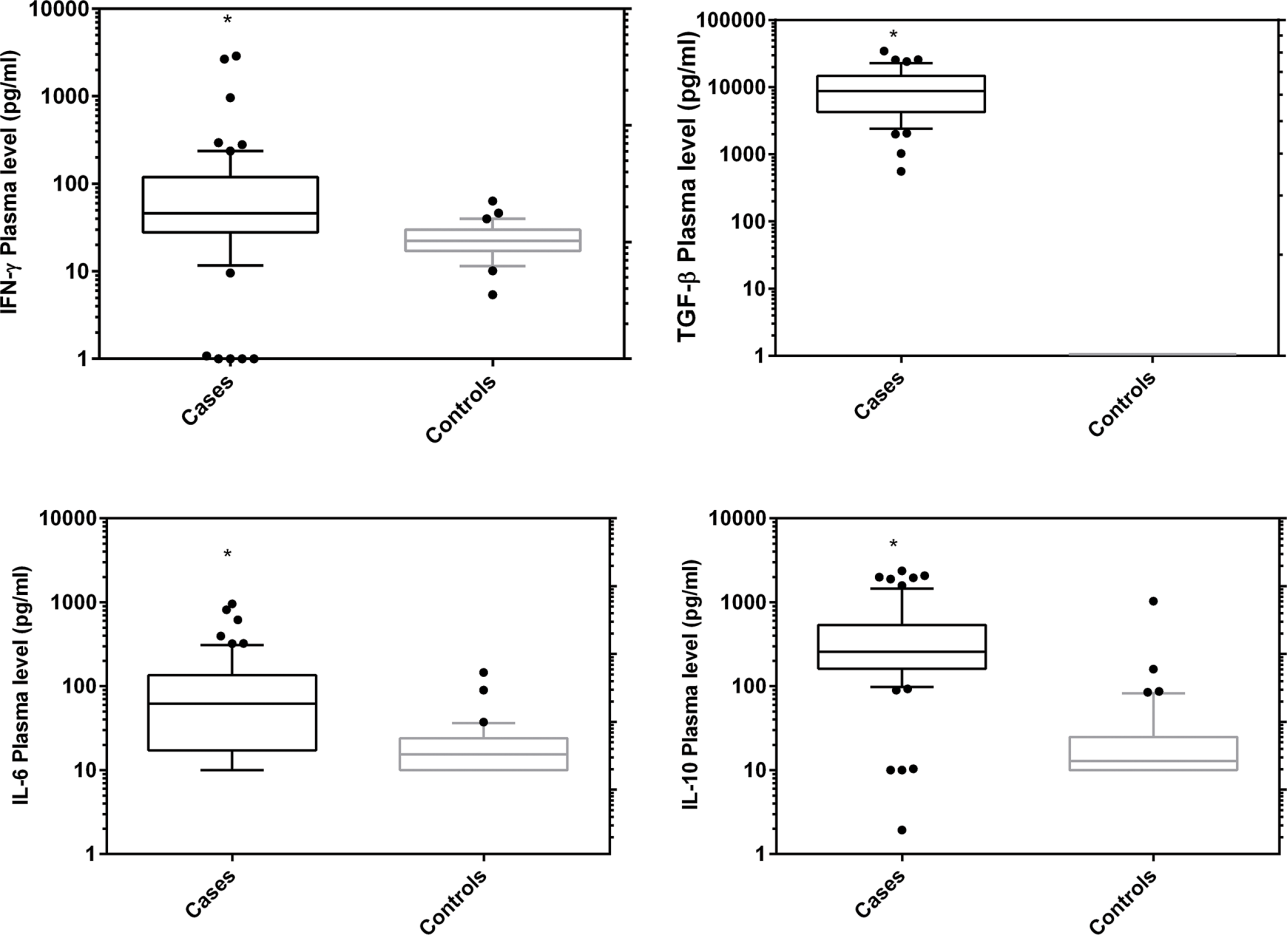
Plasma cytokine profiles in HAT cases (N = 49) and controls (N = 41). Boxes indicate median and interquartile range, whiskers are defined as 10^th^ -90^th^ percentiles. Dots define outliers. Asterisk (*) indicate significant increase in cases over controls (Mann-Whitney U test, P< 0.05).

**Fig 2:**
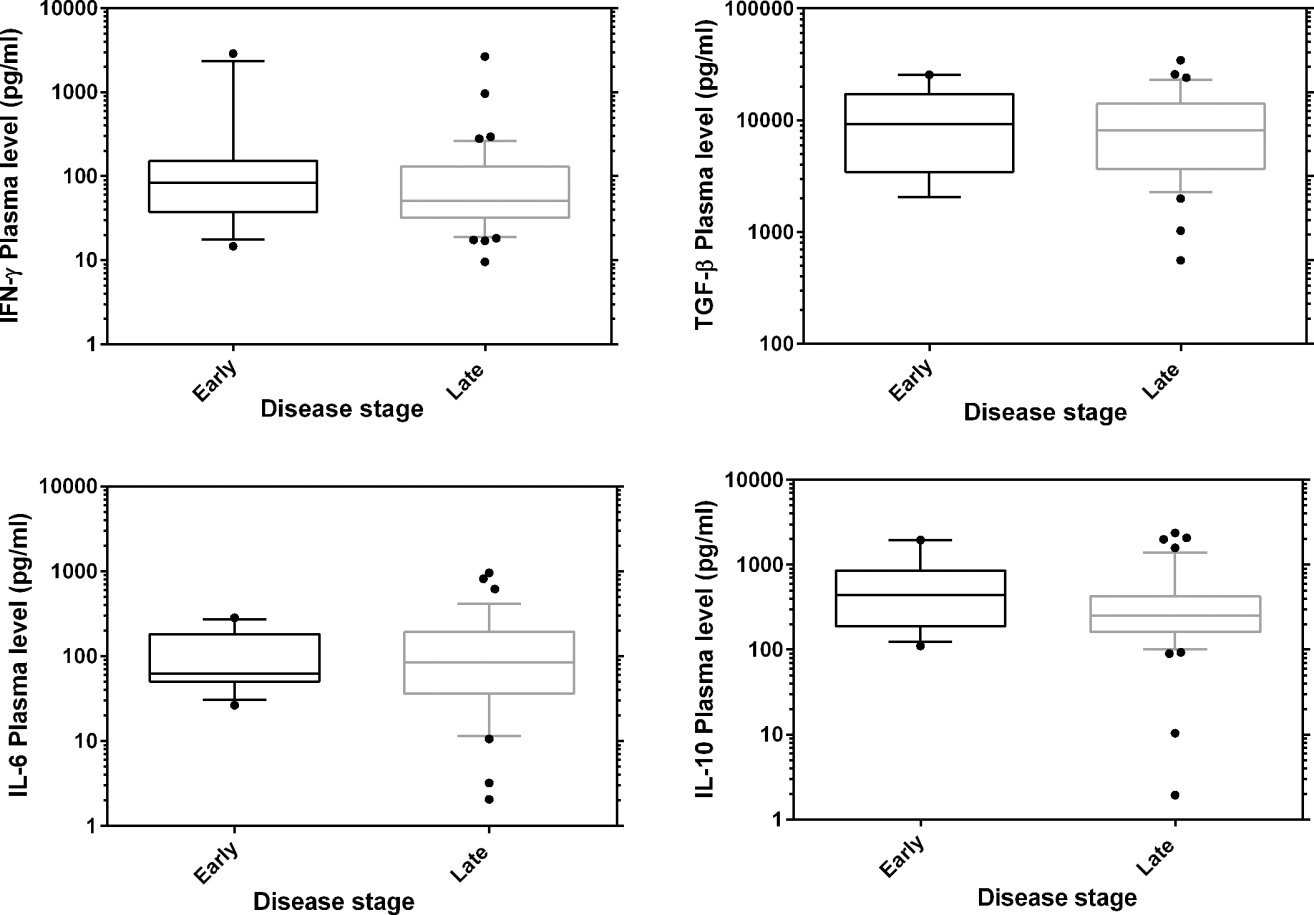
Plasma cytokine profiles in HAT patients compared according to disease stage. Boxes indicate median and interquartile range, whiskers are defined as 10^th^ -90^th^ percentiles. Dots define outliers.

In order to demonstrate the role of cytokines in the modulation of HAT progression, we assayed six cytokines (IFN-γ, IL1-β, TNF-α, IL-6, TGF-β and IL-10) in plasma of both cases (N = 49, after excluding 6 malaria co-infected patients) and controls (N = 41). The detection limits for the assays were 12.1, 6.1, 2.7, 23, 1025 and 14.2 pg/ml respectively. Median plasma levels (pg/ml) of IFN-γ (46.3), IL-6 (61.7), TGF-β (8755) and IL-10 (256.6) were significantly higher in cases compared to controls (Fig 1, (Mann Whitney U test, p< 0.0001)). IL1-β was detected in plasma of one HAT case while TNF-α was only detected in 2 cases and in 2 control samples. When median plasma levels of early stage cases and controls were compared, IFN-γ, IL-6, IL-10 and TGF-β remained significantly elevated over controls (Mann-Whitney U test, P< 0.0001, S1 Fig). Median plasma cytokine concentrations did not differ significantly between early and late stage patients (Fig 2, Man-Whitney U test, P> 0.05). There was a positive correlation between plasma IL-10 and IL-6 (Spearman rho 0.785, p< 0.0001) as well as IL-10 and IFN-γ (Spearman rho 0.27, P< 0.034). There was a strong positive relationship between blood stream parasitemia and IL-6 (Spearman rho 0.677, P< 0.0001) as well as IL-10 (Spearman rho 0.599, P< 0.0001). We associated the plasma cytokine levels with disease presentation, plasma IL-6 levels negatively correlated with splenomegaly (Spearman rho -0.473, P< 0.02, S1 Table). Plasma cytokine levels were not associated with sex, age or reported duration of disease.

### CSF cytokines and stage progression

**Fig 3:**
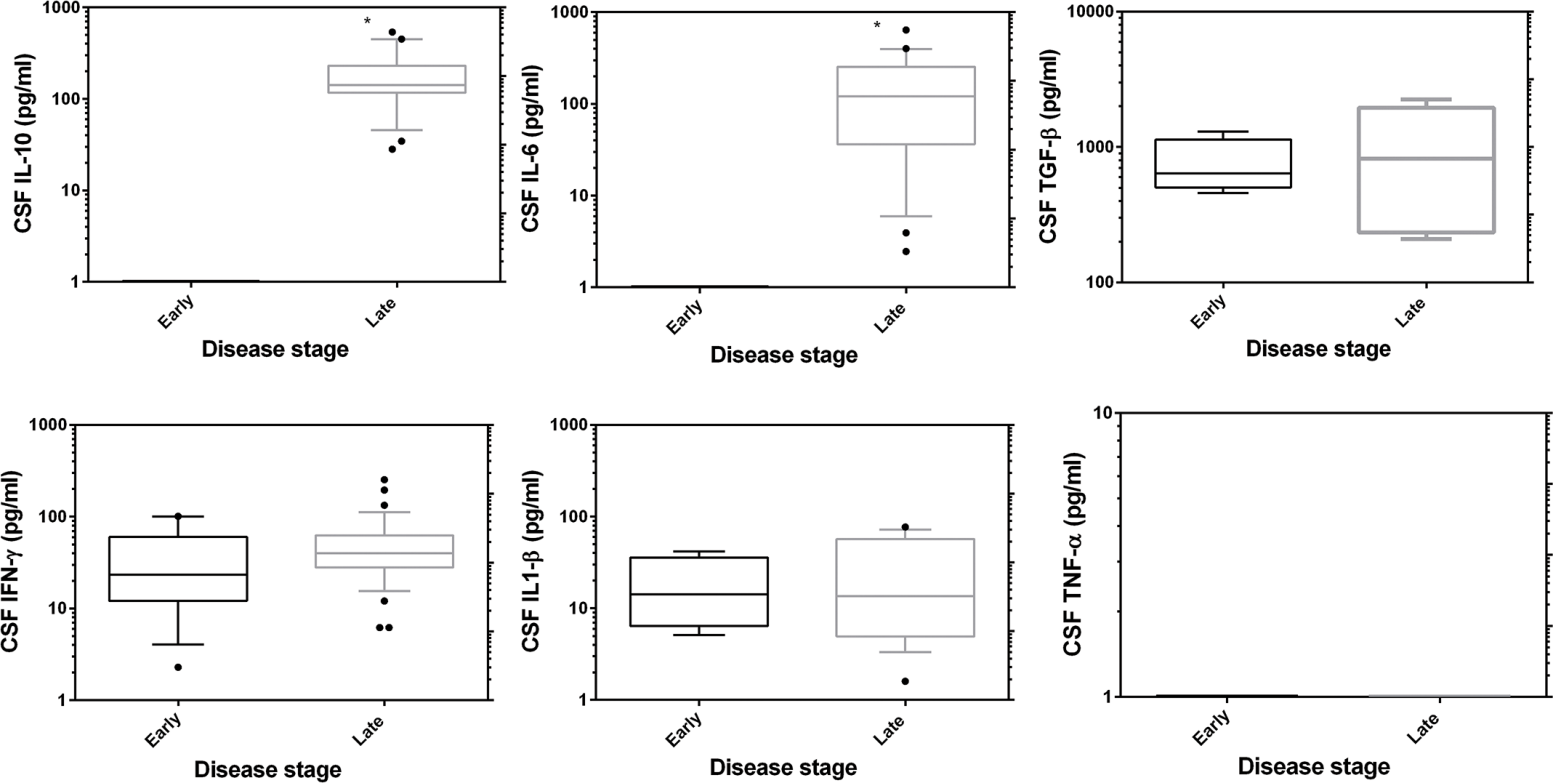
CSF cytokine profiles in HAT patients compared according to disease stage. Boxes indicate median and interquartile range, whiskers are defined as 10^th^ -90^th^ percentiles. Dots define outliers. Asterisk (*) indicate significant increase in late stage patients (Mann-Whitney U test, P< 0.05).

When early stage and late stage cytokines were compared, IL-10 and IL-6 were significantly elevated in late stage patients (Fig 3, Mann-Whitney U test, P< 0.0001). TNF-α was not detected in CSF, while IL1-β, TGF-β and IFN-γ were not associated with disease stage. The presence of trypanosomes in CSF was inversely correlated with levels of IL-6 (Spearman rho -0.494, P< 0.001) and IL-10 (Spearman rho -0.388, P< 0.006). There was a strong positive correlation between IL-6 and IL-10 (Spearman rho 0.634, P< 0.001) and between IFN-γ and IL1-β (Spearman rho 0.443, P< 0.001). Patients with CSF trypanosomes had significantly elevated WBCs (P< 0.000). There was a positive co-relationship between WBC and CSF IL-6 (Spearman rho 0.404, P< 0.004) similarly with IL-10 (Spearman rho 0.289, P< 0.047). We correlated CSF cytokine levels with disease duration; IL-6 (Spearman rho 0.590, P< 0.008) and IL1-β (Spearman rho 0.510, P< 0.026) had a positive relationship. We did not find any association between the degree of brain injury as measured by the Glasgow coma score and CSF cytokine levels, however, IL-10 and IL-6 were inversely associated with tremors (Spearman rho -0.472, P< 0.03 and Spearman rho -0.45, P< 0.04 respectively). Furthermore IL-10 had a significant inverse association with cranioneuropathy (Spearman rho -0.547, p< 0.02, S2 Table).

### IL-6 and IL-10 as potential stage biomarkers

In order to explore the possibility of using IL-6 and IL-10 as possible stage markers, receiver operator characteristic (ROC) curves were analyzed to assess their ability to discriminate between early and late stage patients (S2 Fig). IL-6 and IL-10 had a higher area under curve (AUC) of 0.97 (95% CI, 0.90–1.0) and 0.96 (95% CI, 0.89–1.0) respectively (Table 2). A high staging accuracy for IL-6 was obtained by using a cutoff of > 23.3 pg/ml with a sensitivity of 83.3% (95% CI, 62.2%-95.3%) and a specificity of 100% (95% CI, 47.8%-100%). For IL-10, a specificity of 100% (95% CI, 39.8%-100%) gave a sensitivity of 85.7% (95% CI, 63.7%-97%) with a cutoff of > 61.5 pg/ml.

**Table 2:** Potential of CSF cytokines as stage biomarkers.

Marker	Correlation with presence of parasites	Correlation with WBC, Spearman rho	ROC curve	pAUC	Cut off (pg/ml)	Sensitivity % (95% CI)	Specificity % (95% CI)
IL6	-0.473**	0.404**	0.97	0.001*	>23.3	83.3 (62.2–95.3)	100 (47.8–100)
IL10	-0.388**	0.289**	0.96	0.004*	>61.5	85.7 (63.7–97)	100 (39.8–100)
IFN-γ	-0.154	0.211	0.66	0.09	>50.3	41.7 (25.5–59.2)	76.9 (46.2–95)
IL1-β	0.045	-0.1	0.52	0.92	>18.4	46.7 (21.3–73.4)	75 (19.4–99.4)
TGF-β	0.071	-0.054	0.5	0.9	>838	50 (11.8–88.2)	75 (19.4–99.4)

** Correlation significant at the 0.01 level (2-tailed).

*Correlation significant at the 0.05 level.

## Discussion

In this study we have carried out a comprehensive analysis of cytokines in *T*. *b*. *rhodesiense* HAT patients and analyzed them in light of disease stage, duration and severity. Sleeping sickness due to *T*. *b*. *rhodesiense* was previously described as an acute disease [31]. However, recent studies reporting a wide range of disease pathology with variation in severity and progression both within and across disease foci have started to emerge [3,4]. HAT patients in Uganda (Eastern Africa) were reported to suffer from a more acute disease compared to patients from Malawi (Southern Africa) [3]. Indeed, even when HAT pathology was compared in two geographically close foci in Uganda (Tororo and Soroti) significant differences in disease presentation and progression were noted [5]. This variation in HAT severity and progression has been proposed to be due to variation in host inflammatory cytokines in both human patients [3,5,32] and in experimental animals [11,27,33]. However, controversies still exist about the roles of specific cytokines in HAT progression [7].

Our data shows that plasma concentrations of IFN-γ, TGF-β, IL-6 and IL-10 were higher in patients than in controls as previously described in both *T*. *b*. *rhodesiense* [3,5] and *T*. *b*. *gambiense* patients [34,35]. We did not find a significant difference between early stage and late stage plasma cytokine levels. However, when CSF cytokine levels for early and late stage patients were compared, both IL-6 and IL-10 were up-regulated in late stage patients. No stage differences were observed in CSF concentrations of IL-1β, TGF-β and IFN-γ. Pro-inflammatory cytokines have been proposed to be generators of CNS inflammation and pathology [36]. In experimental animal models, TNF-α, IL-1β and IFN-γ have been associated with neuropathology [10]. TNF-α dysregulation has been associated with blood brain barrier dysfunction and initiation of CNS inflammation and pathology [10,37,38]. In *T*. *b*. *gambiense* disease, high serum levels of TNF-α were correlated with disease severity [39]. In *T*. *b*. *rhodesiense* studies, plasma TNF-α levels were elevated in early stage Ugandan patients but remained at normal levels in the late stage [3]. However, in a study comparing two geographically related HAT foci in Uganda (Tororo and Soroti), TNF-α levels remained at control levels [5]. In this study TNF-α was only detected in plasma of 2 early stage cases and remained undetectable in the CNS. Like previous observations among T. *b*. *rhodesiense* patients [14], IFN-γ was not associated with stage progression. However, this finding was not consistent with other human studies in which IFN-γ levels varied depending on ethnicity and degree of neurological involvement [5] and similarly in mouse models [10]. IL-1β was only detected in plasma of one sample and did not differ across disease stage in CSF. This is in agreement with mouse models in which CSF IL-1β was shown to be expressed constitutively, but not in agreement with [5] in which plasma IL-1β was above control levels.

Several human and experimental animal studies suggest that levels of pro-inflammatory cytokines are down regulated in late stage infection by elevated levels of counter inflammatory cytokines [10,11,29,34,35,40]. Indeed, IL-6 and IL-10 concentrations were up regulated in late stage disease in the present study accounting for the low levels pro-inflammatory cytokines observed. Elevated levels of both IL-6 and IL-10 were associated with reduced neuropathy in experimental animals [10,11,41]. Genetic studies have shown polymorphism in the IL-6 gene to be associated with lower risk of developing disease [42]. Similarly, in this study both IL-6 and IL-10 were inversely associated with neurological signs of tremor and neuropathy. However, IL-6 is a multifunctional cytokine that might double as inflammatory and counter inflammatory [43,44] and hence its exact role in HAT pathogenesis needs to be elucidated further in controlled experimental animal models. TGF-β is a pleotropic cytokine with both inflammatory and immune-modulatory roles depending on its concentration and environment [45]. High levels of plasma TGF-β in Malawi patients was related with a protective role [3], however, in another related study, the TGF-β concentration was not associated with disease severity. Similarly in this study, TGF-β was not associated with stage progression or disease severity.

Disease staging for HAT follows WHO [17] staging guidelines. However, there is lack of consensus about its efficacy and new stage biomarkers are being sought [46]. Among the stage biomarkers, cytokines and chemokines have shown a higher potential [22–24]. In this study both IL-10 and IL-6 were up regulated in late stage patients with a positive relationship with WBC and presence of trypanosomes in CSF. We therefore investigated their potential as stage markers using receiver operator characteristic curves. Our data revealed that both IL-6 and IL-10 were able to discriminate between late and early stage patients as indicated by the AUC, 97% and 96% respectively. For IL-6, a specificity of 100% produced a sensitivity of 83.3% while for IL-10 a specificity of 100% produced a sensitivity of 85.7%. These results are in range as reported previously [23,24] but point to an improved sensitivity compared to a previous study among *T*. *b*. *rhodesiense* patients in eastern Uganda [16]. However, the possibility of their translation into point-of-care tests for stage determination has obvious draw backs. The first concern is that the markers are not 100% sensitive and consequently some late stage patients would be missed leading to wrong treatment choices that could fuel relapses. Secondly, sleeping sickness is present in areas endemic for other tropical diseases [47], in which case cytokine dysregulations and biomarker potential apply to other CNS disorders [48]. The above drawbacks and the need to rely on the invasive collection of CSF by lumber puncture make the direct field application of CSF cytokines challenging.

Our data has a limitation in the sample size, because of the low disease incidence and the fact that most patients are diagnosed as late stage thereby limiting the number of early stage patients for comparison. The symptoms of early stage HAT are rather unspecific, such that patients are treated for other conditions including malaria and typhoid fever or even visit traditional healers; by the time HAT is ruled in, it is already in late stage. According to the Glasgow coma score for assessing the degree of neurological involvement, only one patient was classified with severe neurological impairment, limiting meaningful statistical analysis in this group. However, the generally recognized acuteness of *T*. *b*. *rhodesiense* sleeping sickness might limit observing patients with advanced neurological impairment. Therefore, studies utilizing larger cohorts with active screening to capture early stage patients would be useful.

In conclusion, the present study reinforces previous observations about the role of host pro- and counter inflammatory cytokines in the progression and severity of *T*. *b*. *rhodesiense* sleeping sickness. We show an up regulation of IL-6 and IL-10 during the late stage that is associated with a reduction in severity of neurological involvement. Despite the high staging accuracy shown, IL-6 and IL-10 cannot be used on their own as clear cut stage markers since other CNS infections and disorders other than HAT can lead to such elevations. Therefore, these analysis should be validated in a larger cohort of patients and further evaluated in other inflammatory diseases such as cerebral malaria.

## Supporting Information

S1 Checklist STROBE checklist. (DOC)

S1 Table Relationship between plasma cytokine levels and clinical presentation. (DOCX)

S2 Table Relationship between CSF cytokine levels and neurological signs. (DOCX)

S1 Fig Plasma cytokine profiles compared between early stage patients and controls. Boxes indicate median and interquartile range, whiskers are defined as 10^th^ -90^th^ percentiles. Dots define outliers. Asterisk (*) indicate significant increase in early stage over controls (Mann-Whitney U test, P< 0.05).
(TIF)

S2 Fig Receiver operator characteristic curves for the different cytokines. The Area under the ROC curve (AUC) is shown at a significant level (p< 0.05). The diagonal line indicates the line of identity.
(TIF)
