# Supplementary material for: Interleukin (IL)-6 and IL-10 Are Up Regulated in Late Stage Trypanosoma brucei rhodesiense Sleeping Sickness
Source: PLoS Negl Trop Dis. 2015 Jun 19;9(6):e0003835. doi: 10.1371/journal.pntd.0003835 (PMC4474433; doi:10.1371/journal.pntd.0003835)
Supplement: S1 Checklist — (DOC) [file pntd.0003835.s001.doc]

STROBE Statement—Checklist of items that should be included in reports of ***case-control studies***

|  | Item No | Recommendation |
| --- | --- | --- |
| **Title and abstract** | 1 | **Title**: Interleukin (IL)-6 and IL-10 are Up Regulated in Late Stage *Trypanosoma brucei rhodesiense* Sleeping Sickness |
|  |  | **Abstract:** Sleeping sickness due to *Trypanosoma brucei rhodesiense* has a wide spectrum of clinical presentations coupled with differences in disease progression and severity across East and Southern Africa. The disease progresses from an early (hemo-lymphatic) stage to the late (meningoencephalitic) stage characterized by presence of parasites in the central nervous system. We hypothesized that disease progression and severity of the neurological response is modulated by cytokines. A total of 55 sleeping sickness cases and 41 healthy controls were recruited passively at Lwala hospital, in Northern Uganda. A panel of six cytokines (IFN-γ, IL1-β, TNF-α, IL-6, TGF-β and IL-10) were assayed from paired plasma and cerebrospinal fluid (CSF) samples. Cytokine concentrations were analyzed in relation to disease progression, clinical presentation and severity of neurological responses. Median plasma levels (pg/ml) of IFN-γ (46.3), IL-6 (61.7), TGF-β (8755) and IL-10 (256.6) were significantly higher in cases compared to controls (p< 0.0001). When early stage and late stage CSF cytokines were compared, IL-10 and IL-6 were up regulated in late stage patients and were associated with a reduction in tremors and cranioneuropathy. IL-10 had a higher staging accuracy with a sensitivity of 85.7% (95% CI, 63.7%-97%) and a specificity of 100% (95% CI, 39.8%-100%) while for IL-6, a specificity of 100% (95% CI, 47.8%-100%) gave a sensitivity of 83.3% (95% CI, 62.2%-95.3%). Our study demonstrates the role of host inflammatory cytokines in modulating the progression and severity of neurological responses in sleeping sickness. We here demonstrate an up-regulation of IL-6 and IL-10 during the late stage with a significant potential as adjunct stage biomarkers. |
| Introduction | | |
| Background/rationale | 2 | Human African Trypanosomiasis (HAT) or sleeping sickness is caused by extra-cellular protozoan parasites *T. b. rhodesiense* (East and Southern Africa) and *T. b. gambiense* (West and central Africa). Although an estimated 12.3 million people are at a risk of developing *T. b. rhodesiense* disease, the number of new cases has reduced for the past 4 years to below 200 cases per year (range 110-190). The disease progresses in two stages, the hemo-lymphatic or early stage is characterized by the proliferation of trypanosomes in blood and lymph. The second or late stage is characterized by invasion of trypanosomes in the central nervous system (CNS) and appears after weeks in the typically acute *T. b. rhodesiense* disease or months in the chronic *T. b. gambiense* HAT.  Traditionally, *T. b. rhodesiense* HAT has been classified as acute . Recently a wide spectrum of clinical presentation coupled with differences in disease progression and severity has been observed . This diversity in disease spectrum has been attributed to variation in infecting parasite genotypes and host immunogenetics . Although few studies have been documented about cytokine dysregulation in *T. b. rhodesiense* HAT patients, it is proposed that cytokines might be key players in HAT inflammatory processes . However, contradictions about the role of specific cytokines during HAT progression have been noted . In experimental animal models, high levels of pro-inflammatory cytokines (IFN-γ and TNF-α) have been associated with moderate to severe neuropathy . HAT patients in Eastern Uganda (Tororo) with high IFN-γ concentrations exhibited faster progression to CNS disease with a high frequency of moderate to severe neurological impairment . However, in other studies IFN-γ has been associated with parasite control and resistance to disease . The late stage of disease has been associated with elevated levels of counter inflammatory cytokines in the CNS of both HAT patients and experimental animal models . The few previous studies indicated that IL-10 and IL-6 were up regulated in the late stage and were associated with reduced severity of neuropathology in experimental and natural infections . However in another related study, IL-10 and IL-6 were not associated with neurological severity ; it was thus necessary to further investigate this controversy as we have done in this study.  Since clinical signs of HAT are not specific, disease staging to guide treatment is based on examination of cerebrospinal fluid (CSF) . Early stage patients are treated with suramin and late stage patients with melarsoprol that is associated with a reactive encephalopathy in 10% of the patients resulting in an overall mortality of 5% . Currently the WHO criteria is recommended for staging , with patients having a WBC of ≤ 5 WBC/μl and no trypanosomes in the CSF classified as early stage, while those with greater than 5 WBC/μl or trypanosomes in the CSF are in the late stage. However, for *T. b. gambiense* there is contradictory information about the effectiveness of treating patients with 6-20 WBC/µl as early stage . A number of adjunct biomarkers for late stage including cytokines have been proposed in both experimental animal models and human patients . |
| Objectives | 3 | In this study, we hypothesized that stage progression and severity of neuropathology is modulated by host inflammatory cytokines  **Specific objectives**  1) To compare plasma and CSF cytokine profiles between HAT cases and controls (for CSF early stage patients acted as controls)  2) To correlate cytokine profiles with disease stage progression and severity of neuropathology  3) To determine the potential of cytokines as stage biomarkers |
| Methods | | |
| Study design | 4 | Patients with HAT and non-infected control individuals presenting at Lwala hospital were recruited. After diagnosis and disease staging by lumber puncture, a detailed clinical history was sought from each patient. Physical examination was done by a medical officer and both nonspecific HAT signs and neurological involvement recorded. The degree of neurological involvement was assessed using the Glasgow coma scale (GCS) . Patients with a GCS of 13-15 were classified as mild, while those with a score of 9-12 and ≤8 were classified as moderate and severe impairment respectively. Cytokine concentrations (IFN-γ, IL1-β, TNF-α, IL-6, TGF-β and IL-10) were measured in triplicates from paired plasma and CSF using a solid phase sandwich ELISA (OptEIA, Becton Dickinson, Belgium) |
| Setting | 5 | Patients were recruited passively at Lwala hospital, a sleeping sickness referral center in Northern Uganda (Kaberamaido District) between 2012 and 2014. The hospital serves a large catchment area spanning several districts including Kaberamaido, Dokolo, Alebtong, Kole, Lira and Soroti. These districts are predominantly inhabited by cattle keeping people with traction as the major purpose. Within this region, an estimated 7.9 million people are at a risk of developing *T. b. rhodesiense* sleeping sickness . All samples used in the study were remnants from the routine normal diagnostic procedures required to guide treatment. For cytokine assays, a 5ml blood sample was collected from each patient in EDTA vacutainers. For cerebrospinal fluid analysis, 3-4ml were drawn by lumbar puncture. Data recorded on the clinical form included demographic characteristic, self-reported symptoms, perceived onset of symptoms, clinical presentation of the disease, laboratory findings, treatment schedule and disease outcome. Neurological symptoms such as convulsions, tremors, urinary incontinence, psychotic behavior and sleep disorders were recorded |
| Participants | 6 | Sleeping sickness patients diagnosed at the hospital were included in the study. Patients below 12 years were excluded. Cases were taken as those having a positive blood smear for trypanosomes, or the patient presented with suspicious HAT signs, with trypanosomes demonstrated in CSF after a lumbar puncture. As controls samples, plasma was collected from HAT free individuals consulting at the hospital. Due to ethical considerations, CSF from controls was not obtained. We therefore used CSF from early stage patients as controls for comparisons with late stage patients since in early stage patients the blood brain barrier is intact and hence trypanosomes are not demonstrable in CSF at this time. |
|  |  | (*b*) For matched studies, give matching criteria and the number of controls per case |
| Variables | 7 | **Outcomes**: plasma and CSF cytokines quantified, clinical data from each patient collected  **Exposures**: Natural sleeping sickness infections  **Potential confounders**: Co-infections, especially malaria  **Diagnostic criteria:** Routine diagnosis of suspected HAT patients, was done by microscopic examination of wet and thick blood films from finger prick blood, or using the Heamatocrit Centrifugation Technique. If the blood smear was positive for trypanosomes, or the patient presented with suspicious HAT signs, a lumbar puncture was performed. Late stage infection was confirmed by the presence of trypanosomes in the CSF and/or a white blood cell count > 5/µl |
| Data sources/ measurement | 8* | A detailed clinical history was sought from each patient with patient physical examination done by a medical officer and both nonspecific HAT signs and neurological involvement recorded. A 5ml blood sample was collected from each patient in EDTA vacutainers while for CSF, 3-4ml were drawn by lumbar puncture. Cytokine concentrations (IFN-γ, IL1-β, TNF-α, IL-6, TGF-β and IL-10) were measured in triplicates from paired plasma and CSF using a solid phase sandwich ELISA (OptEIA, Becton Dickinson, Belgium) |
| Bias | 9 | Clinical examination was performed by the same trained medical officer. samples in the laboratory were run in triplicates at all times. |
| Study size | 10 | The sample size required for this study was calculated using GraphPad StatMate 2.00 as that with a significant level (alpha) of 0.05 and a 95% power of detecting a difference between means of 23.7 (MacLean, 2007) between cytokine assays. This gave a sample size of 80 (40 from the patients and 40 from the controls). |
| Quantitative variables | 11 | Data was coded and analysed using IBM SPSS version 22 and sensitivity and specificity analysis done using GraphPad Prism version 6.0 statistical software. Deviation from normality was tested using D'Agostino-Pearson normality test. Because none of the cytokines presented a normal distribution, data was presented as medians |
| Statistical methods | 12 | Comparisons between groups were done using the Mann-Whitney U and Kruskal-Wallis non-parametric tests at a significant level (P< 0.05, 2 tailed). Correlation analysis was done using bivariate non-parametric Spearman correlation test set at a significance level of (P< 0.01 and P< 0.05, 2 tailed tests). To determine the potential of cytokines as late stage markers, receiver operator characteristic (ROC) curves were used to calculate the area under the ROC curve (AUC) with sensitivity and specificity predictions for each marker. Sensitivity and specificity calculations were performed using equations indicated below. Each value in the data set was used as a cut off value and a thresh hold cutoff value selected as that producing the best combination of sensitivity and specificity.  Sensitivity= True Positive/ (True Positive + False Negative)  Specificity= True Negative/ (True Negative + False Positive) |
| Results | | |
| Participants | 13* | A total of 55 patients and 41 healthy controls were recruited. since disease stage for one patient could not be ascertained due to limited amounts of CSF 54 patients were used to compare late stage. Malaria co-infections were detected in 6 HAT cases and subsequently omitted from cytokine and clinical data analysis. Thus living a total of 49 cases for cytokine analysis. |
| Descriptive data | 14* | The sex-ratio (male: female) was 1:3 with a median age for HAT cases of 20 years. Late stage cases were significantly more common (44 out of 54, (P< 0.0001). Disease stage for one patient could not be ascertained due to limited amounts of CSF. During the study period, 1 patient (1.8%) died. Self-reported duration of illness was significantly longer among late stage patients (0.95 months, range 0.25-7, (P< 0.04). |
| Outcome data | 15* | After excluding malaria co-infections, 49 cases were analysed and compared with healthy controls. |
| Main results | 16 | Median plasma levels (pg/ml) of IFN-γ (46.3), IL-6 (61.7), TGF-β (8755) and IL-10 (256.6) were significantly higher in cases compared to controls (Mann Whitney U test, p< 0.0001). IL1-β was detected in plasma of one HAT case while TNF-α was only detected in 2 cases and in 2 control samples. When median plasma levels of early stage cases and controls were compared, IFN-γ, IL-6, IL-10 and TGF-β remained significantly elevated over controls (Mann-Whitney U test, P< 0.0001). Median plasma cytokine concentrations did not differ significantly between early and late stage patients (Man-Whitney U test, P> 0.05). When early stage and late stage cytokines were compared, IL-10 and IL-6 were significantly elevated in late stage patients (Mann-Whitney U test, P< 0.0001). TNF-α was not detected in CSF, while IL1-β, TGF-β and IFN-γ were not associated with disease stage |

| Other analyses | 17 | We did not find any association between the degree of brain injury as measured by the Glasgow coma score and CSF cytokine levels, however, IL-10 and IL-6 were inversely associated with tremors (Spearman rho -0.472, P< 0.03 and Spearman rho -0.45, P< 0.04 respectively). Furthermore IL-10 had a significant inverse association with cranioneuropathy (Spearman rho -0.547, p< 0.02,). In order to explore the possibility of using IL-6 and IL-10 as possible stage markers, receiver operator characteristic (ROC) curves were analyzed. A high staging accuracy for IL-6 was obtained by using a cutoff of > 23.3 pg/ml with a sensitivity of 83.3% (95% CI, 62.2%-95.3%) and a specificity of 100% (95% CI, 47.8%-100%). For IL-10, a specificity of 100% (95% CI, 39.8%-100%) gave a sensitivity of 85.7% (95% CI, 63.7%-97%) with a cutoff of > 61.5 pg/ml. |
| --- | --- | --- |
| Discussion | | |
| Key results | 18 | Our data shows that plasma concentrations of IFN-γ, TGF-β, IL-6 and IL-10 were higher in patients than in controls as previously described in both *T. b. rhodesiense* and *T. b. gambiense* patients . We did not find a significant difference between early stage and late stage plasma cytokine levels. However, when CSF cytokine levels for early and late stage patients were compared, both IL-6 and IL-10 were up-regulated in late stage patients. No stage differences were observed in CSF concentrations of IL-1β, TGF-β and IFN-γ. Pro-inflammatory cytokines have been proposed to be generators of CNS inflammation and pathology . In this study TNF-α was only detected in plasma of 2 early stage cases and remained undetectable in the CNS. Like previous observations among T*. b. rhodesiense* patients , IFN-γ was not associated with stage progression. However, this finding was not consistent with other human studies in which IFN-γ levels varied depending on ethnicity and degree of neurological involvement and similarly in mouse models . IL-1β was only detected in plasma of one sample and did not differ across disease stage in CSF. This is agreement with mouse models in which CSF IL-1β was shown to be expressed constitutively, but not in agreement with in which plasma IL-1β was above control levels.  Several human and experimental animal studies suggest that levels of pro-inflammatory cytokines are down regulated in late stage infection by elevated levels of counter inflammatory cytokines . Indeed IL-6 and IL-10 concentrations were up regulated in late stage disease in the present study accounting for the low levels pro-inflammatory cytokines observed. Elevated levels of both IL-6 and IL-10 were associated with reduced neuropathy in experimental animals . Genetic studies have shown polymorphism in the IL-6 gene to be associated with lower risk of developing disease . Similarly, in this study both IL-6 and IL-10 were inversely associated with neurological signs of tremor and neuropathy. However, IL-6 is a multifunctional cytokine that might double as inflammatory and counter inflammatory and hence its exact role in HAT pathogenesis needs to be elucidated further in controlled experimental animal models. TGF-β is a pleotropic cytokine with both inflammatory and immune-modulatory roles depending on its concentration and environment . High levels of plasma TGF-β in Malawi patients was related with a protective role , however, in another related study, the TGF-β concentration was not associated with disease severity. Similarly in this study, TGF-β was not associated with stage progression or disease severity.  Disease staging for HAT follows WHO staging guidelines. However, there is lack of consensus about its efficacy and new stage biomarkers are being sought . Among the stage biomarkers, cytokines and chemokines have shown a higher potential . In this study both IL-10 and IL-6 were up regulated in late stage patients with a positive relationship with WBC and presence of trypanosomes in CSF. We therefore investigated their potential as stage markers using receiver operator characteristic curves. Our data revealed that both IL-6 and IL-10 were able to discriminate between late and early stage patients as indicated by the AUC, 97% and 96% respectively. For IL-6, a specificity of 100% produced a sensitivity of 83.3% while for IL-10 a specificity of 100% produced a sensitivity of 85.7%. These results are in range as reported previously but point to an improved sensitivity compared to a previous study among *T. b. rhodesiense* patients in eastern Uganda [16]. When used individually, a high specificity cut off would compromise sensitivity and hence misdiagnose late stage patients leading to wrong treatment choices that could fuel relapses. On the other hand, a high sensitivity cut off would expose early stage patients to the unnecessary toxic melarsoprol treatment. |
| Limitations | 19 | Sleeping sickness is present in areas endemic for other tropical diseases , in which case cytokine dysregulations and biomarker potential might apply to other CNS disorders . Our study has a limitation in the number of early stage patients because of the low disease incidence and the fact that most patients are diagnosed as late stage thereby limiting the number of early stage patients for comparison. According to the Glasgow coma score for assessing the degree of neurological involvement, only one patient was classified with severe neurological impairment, limiting meaningful statistical analysis in this group. However, the generally recognized acuteness of *T. b. rhodesiense* sleeping sickness might limit observing patients with advanced neurological impairment. |
| Interpretation | 20 | Like in previous studies, our study demonstrates that cytokines are key modulators in the progression and severity of *T. b. rhodesiense* sleeping sickness. We show an up regulation of IL-6 and IL-10 during the late stage that is associated with a reduction in severity of neurological involvement, indicated that these cytokines might be involved in reducing brain injury caused by presence of trypanosomes. We further reveal that both IL-6 and IL-10 are predictive of late stage involvement with a significant potential as adjunct stage biomarkers. However, it would be interesting to verify their performance with a panel of biomarkers previously identified. |
| Generalisability | 21 | The results of this study are applicable to other sleeping sickness disease foci. our study can also work as a comparison for other infectious disease immunology. |
| Other information | | |
| Funding | 22 | This study was conducted within the framework of the consortium Afrique One “Ecosystem and Population Health: Expanding Frontiers in Health”. Afrique One is funded by the Wellcome Trust (Grant number 087535/Z/08/A, <http://www.afriqueone.net/>). The funders did not have a role in the study design, execution or drafting of the manuscript. |

*Give information separately for cases and controls.

**Note:** An Explanation and Elaboration article discusses each checklist item and gives methodological background and published examples of transparent reporting. The STROBE checklist is best used in conjunction with this article (freely available on the Web sites of PLoS Medicine at http://www.plosmedicine.org/, Annals of Internal Medicine at http://www.annals.org/, and Epidemiology at http://www.epidem.com/). Information on the STROBE Initiative is available at http://www.strobe-statement.org.

1. Simarro PP, Cecchi G, Franco JR, Paone M, Diarra A, Ruiz-Postigo JA, et al. Estimating and mapping the population at risk of sleeping sickness. PLoS Negl Trop Dis. 2012; 6: e1859.

2. Welburn SC, Fèvre EM, Coleman PG, Odiit M,Maudlin I. Sleeping sickness: a tale of two diseases. Trends Parasitol. 2001; 17: 19-24.

3. MacLean L, Chisi JE, Odiit M, Gibson WC, Ferris V, Picozzi K, et al. Severity of human African trypanosomiasis in East Africa is associated with geographic location, parasite genotype, and host inflammatory cytokine response profile. Infect Immun. 2004; 72: 7040-7044.

4. MacLean LM, Odiit M, Chisi JE, Kennedy PG,Sternberg JM. Focus–Specific Clinical Profiles in Human African Trypanosomiasis Caused by Trypanosoma brucei rhodesiense. PLoS Negl Trop Dis. 2010; 4: e906.

5. MacLean, Odiit M, MacLeod A, Morrison L, Sweeney L, Cooper A, et al. Spatially and genetically distinct African Trypanosome virulence variants defined by host interferon-γ response. J Infect Dis. 2007; 196: 1620-1628.

6. Sternberg JM,MacLean L. A spectrum of disease in human African trypanosomiasis: the host and parasite genetics of virulence. Parasitology. 2010; 137: 2007-2015.

7. Kennedy PG. Cytokines in central nervous system trypanosomiasis: cause, effect or both? Trans R Soc Trop Med Hyg. 2009; 103: 213-214.

8. Hertz CJ, Filutowicz H,Mansfield JM. Resistance to the African trypanosomes is IFN-γ dependent. J Immunol. 1998; 161: 6775-6783.

9. Russell KL, Ming JE, Patel K, Jukofsky L, Magnusson M,Krantz ID. human and rodent interferon-as a growth factor for trypanosoma brucei. Eur J Immunol. 2005.

10. Sternberg JM, Rodgers J, Bradley B, MacLean L, Murray M,Kennedy PG. Meningoencephalitic African trypanosomiasis: brain IL-10 and IL-6 are associated with protection from neuro-inflammatory pathology. J Neuroimmunol. 2005; 167: 81-89.

11. Namangala B, Noël W, De Baetselier P, Brys L,Beschin A. Relative contribution of interferon-γ and interleukin-10 to resistance to murine African trypanosomosis. J Infect Dis. 2001; 183: 1794-1800.

12. Yamey G. Research Ethics and Reporting Standards at <italic>PLoS Neglected Tropical Diseases</italic>. PLoS Negl Trop Dis. 2007; 1: e69.

13. MacLean L, Odiit M,Sternberg JM. Intrathecal cytokine responses in Trypanosoma brucei rhodesiense sleeping sickness patients. Trans R Soc Trop Med Hyg 2006; 100: 270-275.

14. MacLean L, Reiber H, Kennedy PG,Sternberg JM. Stage progression and neurological symptoms in Trypanosoma brucei rhodesiense sleeping sickness: role of the CNS inflammatory response. PLoS Negl Trop Dis. 2012; 6: e1857.

15. Chappuis F, Loutan L, Simarro P, Lejon V,Büscher P. Options for field diagnosis of human African trypanosomiasis. Clin Microbiol Rev. 2005; 18: 133-146.

16. Kennedy PG. Diagnostic and neuropathogenesis issues in human African trypanosomiasis. Int J Parasitol. 2006; 36: 505-512.

17. WHO. Control and surveillance of African trypanosomiasis: report of a WHO expert committee. WHO Tech Rep Ser. 2013; 984: 1-250.

18. Balasegaram M, Harris S, Checchi F, Hamel C,Karunakara U. Treatment outcomes and risk factors for relapse in patients with early-stage human African trypanosomiasis (HAT) in the Republic of the Congo. Bull World Health Organ. 2006; 84: 777-782.

19. Doua F, Miezan T, Sanon SJ, Boa YF,Baltz T. The efficacy of pentamidine in the treatment of early-late stage Trypanosoma brucei gambiense trypanosomiasis. Am J Trop Med Hyg. 1996; 55: 586-588.

20. Ruiz JA, Simarro PP,Josenando T. Control of human African trypanosomiasis in the Quicama focus, Angola. Bull World Health Organ. 2002; 80: 738-745.

21. Hainard A, Tiberti N, Robin X, Lejon V, Ngoyi DM, Matovu E, et al. A combined CXCL10, CXCL8 and H-FABP panel for the staging of human African trypanosomiasis patients. PLoS Negl Trop Dis. 2009; 3: e459.

22. Ngotho M, Kagira J, Jensen H, Karanja S, Farah I,Hau J. Immunospecific immunoglobulins and IL‐10 as markers for Trypanosoma brucei rhodesiense late stage disease in experimentally infected vervet monkeys. Trop Med Int Health. 2009; 14: 736-747.

23. Tiberti N, Matovu E, Hainard A, Enyaru JC, Lejon V, Robin X, et al. New biomarkers for stage determination in Trypanosoma brucei rhodesiense sleeping sickness patients. Clin Transl Med. 2013; 2: 1.

24. Lejon V, Roger I, Ngoyi DM, Menten J, Robays J, N'Siesi FX, et al. Novel markers for treatment outcome in late-stage Trypanosoma brucei gambiense trypanosomiasis. Clin Infect Dis. 2008; 47: 15-22.

25. Rodgers J. Trypanosomiasis and the brain. Parasitology. 2010; 137: 1995-2006.

26. Teasdale G,Jennett B. Assessment of coma and impaired consciousness: a practical scale. The Lancet. 1974; 304: 81-84.

27. Lejon V, Lardon J, Kenis G, Pinoges L, Legros D, Bisser S, et al. Interleukin (IL)-6, IL-8 and IL-10 in serum and CSF of Trypanosoma brucei gambiense sleeping sickness patients before and after treatment. Trans R Soc Trop Med Hyg. 2002; 96: 329-333.

28. Rhind SG, Sabiston BH, Shek PN, Buguet A, Muanga G, Stanghellini A, et al. Effect of melarsoprol treatment on circulating IL-10 and TNF-α levels in human African trypanosomiasis. Clin Immunol. 1997; 83: 185-189.

29. Kennedy PG. The continuing problem of human African trypanosomiasis (sleeping sickness). Ann Neurol. 2008; 64: 116-126.

30. MacLean L, Odiit M,Sternberg JM. Nitric oxide and cytokine synthesis in human African trypanosomiasis. J Infect Dis. 2001; 184: 1086-1090.

31. Ngotho M, Maina N, Kagira J, Royo F, Farah IO,Hau J. IL-10 is up regulated in early and transitional stages in vervet monkeys experimentally infected with Trypanosoma brucei rhodesiense. Parasitol Int. 2006; 55: 243-248.

32. Penkowa M, Giralt M, Lago N, Camats J, Carrasco J, Hernández J, et al. Astrocyte-targeted expression of IL-6 protects the CNSagainst a focal brain injury. Exp Neuro. 2003; 181: 130-148.

33. Courtin D, Milet J, Jamonneau V, Yeminanga CS, Kumeso VKB, Bilengue CMM, et al. Association between human African trypanosomiasis and the IL6gene in a Congolese population. Infect Genet Evol. 2007; 7: 60-68.

34. Van Wagoner NJ, Oh J-W, Repovic P,Benveniste EN. Interleukin-6 (IL-6) production by astrocytes: autocrine regulation by IL-6 and the soluble IL-6 receptor. J Neurosci. 1999; 19: 5236-5244.

35. Oh J-W, Van Wagoner NJ, Rose-John S,Benveniste EN. Role of IL-6 and the soluble IL-6 receptor in inhibition of VCAM-1 gene expression. J Immunol. 1998; 161: 4992-4999.

36. Wahl SM, McCartney-Francis N,Mergenhagen SE. Inflammatory and immunomodulatory roles of TGF-β. Immunol Today. 1989; 10: 258-261.

37. Kennedy P. Difficulties in diagnostic staging of human African trypanosomiasis. J Neuroparasitol. 2011; 2: 1-3.

38. Kato CD, Nanteza A, Mugasa C, Edyelu A, Matovu E,Alibu VP. Clinical Profiles, Disease Outcome and Co-Morbidities among T. b. rhodesiense Sleeping Sickness Patients in Uganda. PloS one. 2015; 10: e0118370.

39. Guimarães da Costa A, do Valle Antonelli LR, Augusto Carvalho Costa P, Paulo Diniz Pimentel J, Garcia NP, Monteiro Tarragô A, et al. The Robust and Modulated Biomarker Network Elicited by the Plasmodium vivax Infection Is Mainly Mediated by the IL-6/IL-10 Axis and Is Associated with the Parasite Load. J Immunol Res. 2014; 2014.
