# Supplementary material for: Interleukin (IL)-6 and IL-10 Are Up Regulated in Late Stage Trypanosoma brucei rhodesiense Sleeping Sickness
Source: PLoS Negl Trop Dis. 2015 Jun 19;9(6):e0003835. doi: 10.1371/journal.pntd.0003835 (PMC4474433; doi:10.1371/journal.pntd.0003835)
Supplement: S1 Table — (DOCX) [file pntd.0003835.s002.docx]

**S1 Table**. Relationship between plasma cytokine levels and clinical presentation.

| Clinical presentation | IFN-γ | TGF-β | IL-6 | IL-10 |
| --- | --- | --- | --- | --- |
| Fever | - | - | - | - |
| Headache | 0.371 | 0.411 | -0.011 | 0.067 |
| Edema | 0.011 | 0.359 | 0.021 | -0.157 |
| Ascites | 0.340 | -0.294 | -0.085 | 0.045 |
| Hepatomegaly | -0.137 | 0.120 | -0.327 | -0.023 |
| Splenomegaly | -0.125 | 0.246 | -0.473* | -0.238 |
| Lymphadenopathy | 0.116 | -0.079 | 0.001 | 0.063 |
| Somnolence | -0.045 | 0.001 | 0.023 | -0.049 |
| Gait abnormalities | 0.139 | 0.148 | 0.320 | -0.086 |
| Tremors | 0.022 | 0.072 | 0.109 | 0.326 |
| Urinary incontinence | -0.057 | -0.236 | 0.014 | 0.015 |
| Cranioneuropathy | 0.360 | 0.273 | 0.222 | 0.055 |
|  |  |  |  |  |

Correlations coefficients marked with an asterisk (*) were significant at *p* < .05.
