# Supplementary material for: Interleukin (IL)-6 and IL-10 Are Up Regulated in Late Stage Trypanosoma brucei rhodesiense Sleeping Sickness
Source: PLoS Negl Trop Dis. 2015 Jun 19;9(6):e0003835. doi: 10.1371/journal.pntd.0003835 (PMC4474433; doi:10.1371/journal.pntd.0003835)
Supplement: S2 Table — (DOCX) [file pntd.0003835.s003.docx]

**S2 Table**. Relationship between CSF cytokine levels and neurological signs

| Neurological sign | IFN-γ | IL-1β | TGF-β | IL-6 | IL-10 |
| --- | --- | --- | --- | --- | --- |
| Somnolence | 0.143 | -0.399 | -0.415 | -0.065 | 0.109 |
| Gait abnormalities | 0.078 | -0.194 | -0.127 | 0.410 | 0.321 |
| Tremors | -0.174 | 0.228 | 0.165 | -0.45* | -0.472* |
| Urinary incontinence | -0.271 | 0.226 | 0.165 | -0.226 | -0.339 |
| Cranioneuropathy | -0.215 | 0.156 | 0.121 | -0.186 | -0.547* |
|  |  |  |  |  |  |

Correlations coefficients marked with an asterisk (*) were significant at *p* < .05.
